# Wedge resection plus adequate lymph nodes resection is comparable to lobectomy for small-sized non-small cell lung cancer

**DOI:** 10.3389/fonc.2022.1022904

**Published:** 2022-11-11

**Authors:** Hongdou Ding, Nan Song, Peng Zhang, Gening Jiang, Haifeng Wang

**Affiliations:** Department of Thoracic Surgery, Shanghai Pulmonary Hospital, Tongji University School of Medicine, Shanghai, China

**Keywords:** lobectomy, wedge resection, non-small cell lung cancer, lymph nodes resection, overall surivival

## Abstract

**Objectives:**

The study investigated whether wedge resection plus adequate lymph nodes resection conferred comparable survival to lobectomy for node-negative non-small cell lung cancer (NSCLC) ≤2 cm.

**Methods:**

The Surveillance, Epidemiology, and End Results database was used to identify patients diagnosed with node-negative NSCLC ≤2 cm and underwent wedge resection or lobectomy (2004-2015). Patients were stratified by the procedure (wedge resection, lobectomy) and the size of NSCLC (≤1 cm, 1-2 cm). We assessed survival between patients undergoing wedge resection and lobectomy. The optimal number of lymph nodes resected which made those two procedures comparable was explored by using Kaplan-Meier analysis and Cox regression analysis. Propensity score matching was performed to minimize the effect of confounding factors.

**Results:**

7893 patients with lobectomy and 2536 patients with wedge resection were identified. Wedge resection was associated with worse survival either in the ≤1 cm or 1-2 cm NSCLC before and after matching. For lesions 1-2 cm and receiving lobectomy, more lymph nodes resected conferred statistically significant increase on survival and six nodes were optimal. For lesions ≤1 cm and receiving lobectomy, lymph nodes resection had no impact on survival. Wedge resection and lobectomy were comparable when one or more nodes for lesions ≤1 cm and six or more nodes for lesions 1-2 cm were resected.

**Conclusions:**

Wedge resection was inferior to lobectomy for NSCLC ≤1 cm and 1-2 cm. Wedge resection plus adequate lymph nodes resection was comparable to lobectomy.

## Highlights:

1. Wedge resection was inferior to lobectomy for node-negative NSCLC ≤1 cm or 2 cm in the population.

2. Wedge resection plus one or more nodes resected for NSCLC ≤1 cm was comparable to lobectomy.

3. Wedge resection plus six or more nodes resected for NSCLC 1-2 cm was comparable to lobectomy.

## Introduction

Accounting for more than 80% of lung cancer, non-small cell lung cancer (NSCLC) is becoming the most cancer-related deaths around the world (1). Owing to the spread of screening by computed tomography (CT), more and more small-sized NSCLCs are detected and diagnosed. For those early-staged lung cancer, surgery is the standard treatment of care and confers a favorable prognosis. Besides lobectomy, sublobar resection had been an important choice because of its minimal invasiveness and preservation of more lung function. Prior studies have compared the oncological outcomes of lobectomy and sublobar resection and exhibited contradictory results. For the population, sublobar resection seems to carry less survival benefit versus lobectomy (2, 3). However, when not selected as compromised procedure or for screen-detected lung cancer, sublobar resection is comparable to lobectomy (4).

Comprising approximately 80% of sublobar resection, wedge resection is more frequently performed compared to segmentectomy (5). Sampling lymph nodes in remaining pulmonary lobes is difficult and thus it is challenging to achieve complete lymph nodes resection for wedge resection. The necessity of lymph nodes resection and the exact number of examined nodes are questionable in consideration of its clinical benefit. Our previous study demonstrated that the number of resected lymph nodes during wedge resection significantly impact the overall survival among patients with node-negative NSCLC ≤2 cm (5). Herein, we compared the survival among patients with NSCLC ≤2 cm and receiving wedge resection or lobectomy after propensity score matching. Furthermore, we explored whether wedge resection plus adequate lymph node resection conferred comparable long-term outcomes to lobectomy in the population.

## Methods

### Study population

The retrospective study was conducted by using the Surveillance, Epidemiology, and End Results (SEER) database. The characteristics of the SEER database have been well described (6, 7). Briefly, the SEER contains cases from more than 20 geographically different registries and covers approximately 48% of the population of the United States. We selected eligible patients by using the following inclusion criteria (1): pathologically confirmed NSCLC, (2) diagnosed between 2004 and 2015, (3) tumor size ≤2 cm (T1a or T1b), (4) lobectomy (code 30, 33) or wedge resection (code 21) performed, and (5) NSCLC as the only primary tumor until the end of follow-up period. We excluded patients with (1) unknown number of examined lymph nodes, (2) nodal disease, (3) distant metastasis (M1a or M1b), (4) follow-up time less than 2 months.

### Data collection

Demographic variables (age, sex, race, and marital status), tumor characteristics (size, histologic type, site), and treatment information (surgical procedure and number of examined lymph nodes) were collected. Histologic types of NSCLC were categorized as adenocarcinoma (codes 8140, 8230, 8240, 8250, 251, 8252, 8253, 8254, 8255, 8260, 8310, 8333, 8470, 8480, 8481, 8490, 8550), squamous carcinoma (codes 8052, 8070, 8071, 8072, 8073, 8083, 8084) and other lung cancer (codes 8012, 8013, 8014, 8031, 8560, 8046). Survival time was retrieved from the SEER record. The database offered details on whether each patient survived or died due to lung cancer or other causes at the end of the last follow-up. Overall survival (OS) and cause-specific survival (CSS) were the primary end point for the study.

### Statistical analysis

To minimize the effect of potential confounding factors, propensity score matching (method =“nearest”, ratio=1:1) was performed for variables including sex, age, race, marital status, histologic type, and tumor site. To evaluate the effect of lymph node resection on survival in NSCLC undergoing lobectomy, we compared survival of cases with different number of resected lymph nodes with those without lymph nodes resection. The cutoff number of resected lymph nodes which conferred wedge resection non-inferior survival than lobectomy was determined as following: cases with more than a certain number of lymph nodes resected (starting from 0) were selected and after performing propensity score matching, survival was assessed between wedge resection group and lobectomy group until the difference did not reach statistical significance. The number of removed nodes was not included in the matching, as the previous study did (8).

Baseline characteristics were compared using independent sample *t* test for continuous variables and Pearson χ^2^ test or Fisher’s exact test for categorical variables. Cases were stratified by tumor size and generated two subgroups: 0-1 cm and 1-2 cm. Locally estimated scatterplot smoothing (LOESS) was performed to generate a smooth curve to describe the association between the number of resected lymph nodes and corresponding hazard ratio (HR). Kaplan-Meier method was used to estimate the association between the number of lymph nodes examined and survival by log-rank test. Cox proportional hazards modeling was performed to determine the potential effect of the number of lymph nodes examined on survival, with adjustment for sex, age, race, marital status, histologic type, and tumor site. All analyses were performed with R Statistical Software (version 4.1.1; Vienna, Austria). We considered two-sided *p* less than 0.05 as statistical significance. Cases were filtered and their corresponding information was obtained by using SEER*Stat version 8.3.9 software.

## Results

### Population characteristics

**Figure 1:**
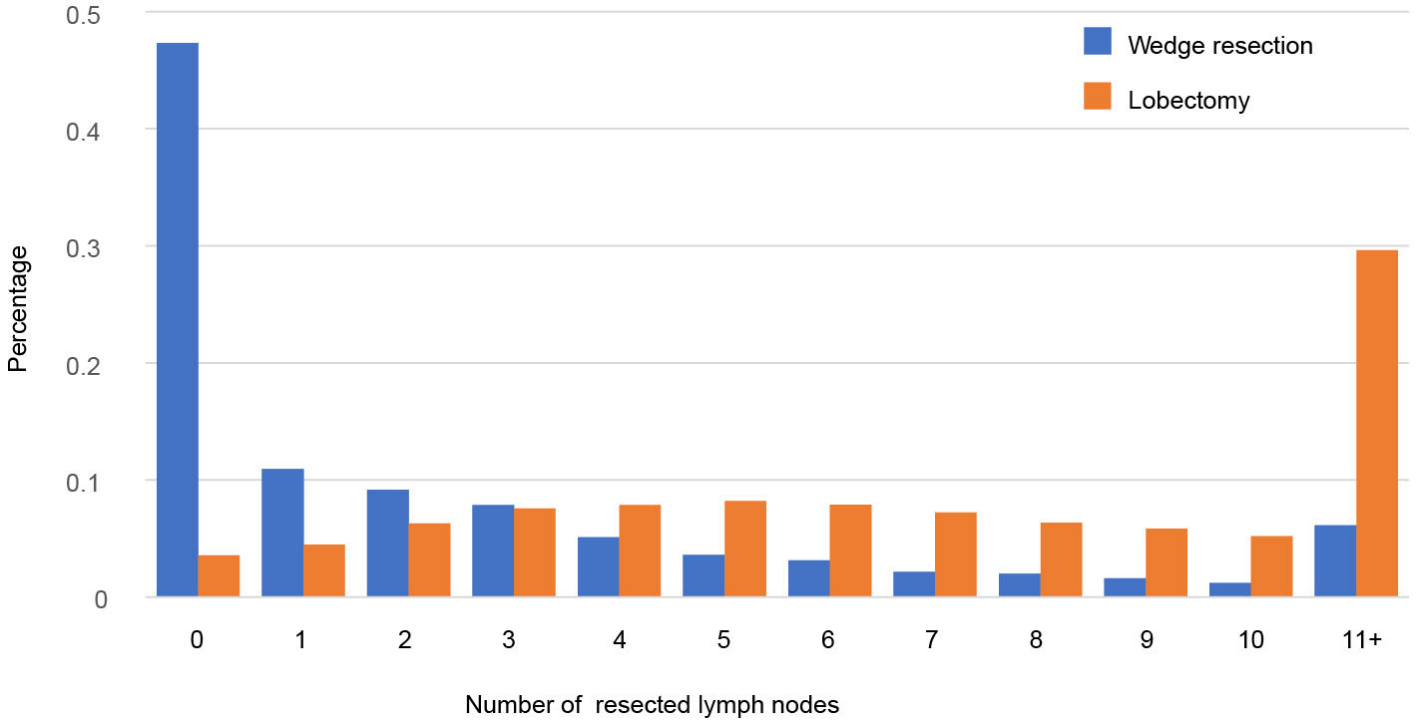
Distribution of the number of examined lymph nodes for wedge resection and lobectomy.

Totally, 10429 patients were extracted, including 7893 patients undergoing lobectomy and 2536 undergoing wedge resection (**Table 1**). The majority of patients were female (59.4%), >65 years old (61.1%), white race (85.2%), married (55.7%) and pathological confirmed adenocarcinoma (72.3%). There were differences in age, race, marital status, histologic type, grade and tumor site between lobectomy group and wedge resection group. Tumors undergoing wedge resection have smaller size and less lymph nodes resected. The proportional distributions of resected nodes in both groups are showed in **Figure 1**. Nearly 47.3% of tumors undergoing wedge resection had no nodes resected, while 3.5% of those undergoing lobectomy did. The ratio of cases with more than 10 nodes resected in wedge resection was much lower than that in lobectomy (6.1% *vs.* 29.6%).

**Table 1:** Baseline characteristics of patients with NSCLC 2 cm or less undergoing wedge resection or lobectomy (n=10429).

Characteristic	Overall (n = 10429)	Lobectomy (n = 7893, %)	Wedge resection (n = 2536,%)	*p* value
Sex, female	6195 (59.4)	4705 (59.6)	1490 (58.8)	0.459
Age, >65 years	6375 (61.1)	4608 (58.4)	1767 (69.7)	<0.001
Race, nonwhite	1545 (14.8)	1212 (15.4)	333 (13.1)	0.007
Marriage				0.032
Married	5808 (55.7)	4452 (56.4)	1356 (53.5)	
Unmarried	4216 (40.4)	3143 (39.8)	1073 (42.3)	
Unknown	405 (3.9)	298 (3.8)	107 (4.2)	
Histologic type				<0.001
Adenocarcinoma	7541 (72.3)	5839 (74.0)	1702 (67.1)	
Squamous carcinoma	2178 (20.9)	1548 (19.6)	630 (24.8)	
Others	710 (6.8)	506 (6.4)	204 (8.1)	
Grade				<0.001
Well differentiated	2500 (24.0)	1870 (23.7)	630 (24.8)	
Moderately differentiated	4614 (44.2)	3590 (45.5)	1024 (40.4)	
Poorly differentiated	2460 (23.6)	1820 (23.1)	640 (25.2)	
Undifferentiated	109 (1.0)	75 (1.0)	34 (1.3)	
Unknown	746 (7.2)	538 (6.8)	208 (8.2)	
Site				0.001
Upper lobe	6573 (63.0)	4935 (62.5)	1638 (64.6)	
Middle lobe	599 (5.8)	494 (6.3)	105 (4.1)	
Lower lobe	3144 (30.1)	2380 (30.2)	764 (30.1)	
Others	113 (1.1)	84 (1.1)	29 (1.1)	
Tumor size, mean ± SD, cm	1.47 ± 0.39	1.51 ± 0.39	1.38 ± 0.42	<0.001
Lymph nodes examined, median (IQR)	6 (2-10)	7 (4-12)	1 (0-3)	<0.001
Follow-up time, median (range), months	65 (2-179)	69 (2-179)	57 (2-179)	<0.001

IQR, interquartile range; NSCLC, non-small cell lung cancer.

### Survival of lobectomy and wedge resection

**Figure 2:**
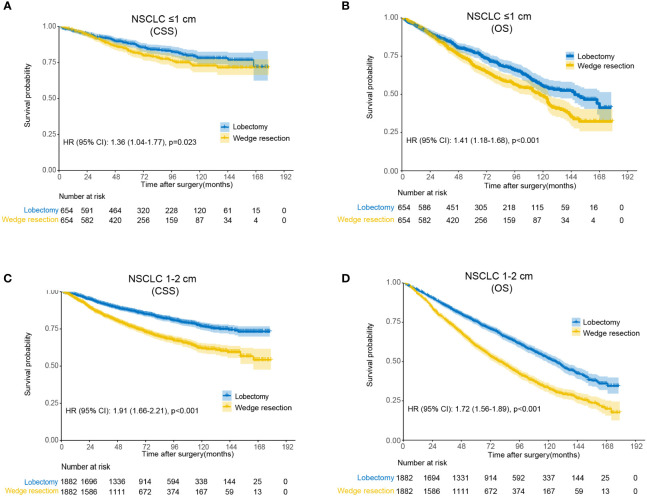
Kaplan-Meier analysis of survival between wedge resection and lobectomy after matching. **(A)** Cause-specific survival for non-small cell lung cancer (NSCLC) ≤1 cm; **(B)** overall survival for NSCLC ≤1 cm; **(C)** cause-specific survival for NSCLC 1-2 cm; **(D)** overall survival for NSCLC 1-2 cm.

Compared to wedge resection, the superiority of lobectomy for CSS and OS was determined in both tumors ≤1 cm and 1-2 cm in diameter (**Supplemental Figure 1**). After propensity score matching, clinicopathological features of both groups were well balanced (**Supplemental Table 1**). Multivariable Cox regression analysis revealed that wedge resection conferred worse OS than lobectomy regarding of the tumor size (0-1 cm: HR, 1.41, 95% CI: 1.18-1.68; 1-2 cm: HR, 1.72; 95% CI: 1.56-1.89). The results were similar when CSS were analyzed (**Figure 2**).

### Survival and number of resected nodes in lobectomy

**Figure 3:**
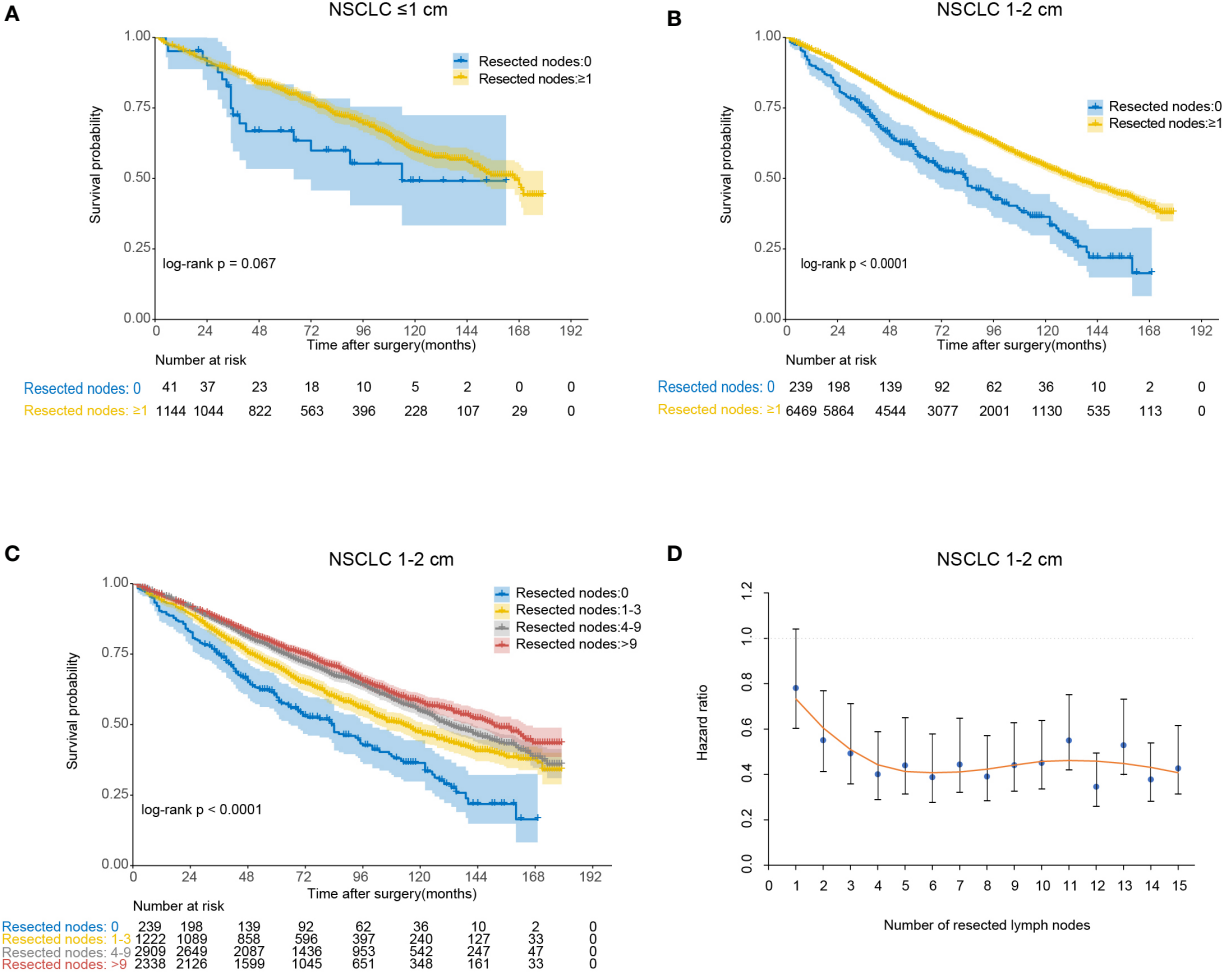
The association between the number of resected lymph nodes and overall survival for lobectomy. **(A)** Kaplan-Meier analysis of overall survival between no lymph nodes resected group and lymph nodes resected group for non-small cell lung cancer (NSCLC) ≤1 cm; **(B)** Kaplan-Meier analysis of overall survival between no lymph nodes resected group and lymph nodes resected group for NSCLC 1-2 cm; **(C)** Kaplan-Meier analysis of overall survival among different lymph nodes resected groups (0, 1-3, 4-9 and >9) for NSCLC 1-2 cm; **(D)** Locally estimated scatterplot smoothing (LOESS) curve describing the association between the specified number of resected lymph nodes and corresponding hazard ratio (HR) for NSCLC 1-2 cm.

We compared OS in patients undergoing lobectomy with or without lymph nodes resection (**Figure 3**). For lesions ≤1 cm, patients derived no significant survival benefit from nodes resection (*log-rank p*=0.067). For lesions 1-2 cm, there was a remarkable increase of OS in patients with nodes resection (*log-rank p*<0.001). According to the number of resected nodes, we subclassified patients to four subgroups as our prior study did (5): 0 nodes, 1-3 nodes, 4-9 nodes and >9 nodes. On multivariable Cox regression analysis, a trend towards more favorable OS and CSS was observed in those who received more lymph nodes resected (**Table 2** and **Supplemental Table 2**). When comparing the OS of cases with a specific number of nodes resected to those without any lymph nodes resected, the survival benefit elevated along with the increase of examined nodes number and peaked when 6 nodes resected as shown by the LOESS curve (**Figure 3D** and **Supplemental Table 3**). More than 6 nodes resected seemed not to generate additional survival benefit.

**Table 2:** Univariable and multivariable analysis of overall survival among patients undergoing lobectomy with different number of lymph nodes resected (0 as reference).

Size		Univariable analysis		Multivariable analysis	
		HR (95% CI)	*p* value	HR (95% CI)	*p* value
0-1 cm					
	≥1 nodes	0.64 (0.39-1.04)	0.637	0.62 (0.37-1.01)	0.056
	1-3 nodes	0.71 (0.42-1.20)	0.205	0.67 (0.39-1.15)	0.144
	4-9 nodes	0.60 (0.36-0.99)	0.047	0.60 (0.36-1.00)	0.050
	≥10 nodes	0.64 (0.39-1.06)	0.086	0.60 (0.36-1.02)	0.057
1-2 cm					
	≥1 nodes	0.54 (0.46-0.64)	<0.001	0.60 (0.51-0.72)	<0.001
	1-3 nodes	0.66 (0.55-0.80)	<0.001	0.75 (0.62-0.91)	0.003
	4-9 nodes	0.53 (0.45-0.64)	<0.001	0.59 (0.50-0.71)	<0.001
	≥10 nodes	0.48 (0.40-0.57)	<0.001	0.53 (0.44-0.63)	<0.001

CI, confidence interval; HR, hazard ratio.

### Survival of wedge resection/lobectomy with adequate nodes resected

**Figure 4:**
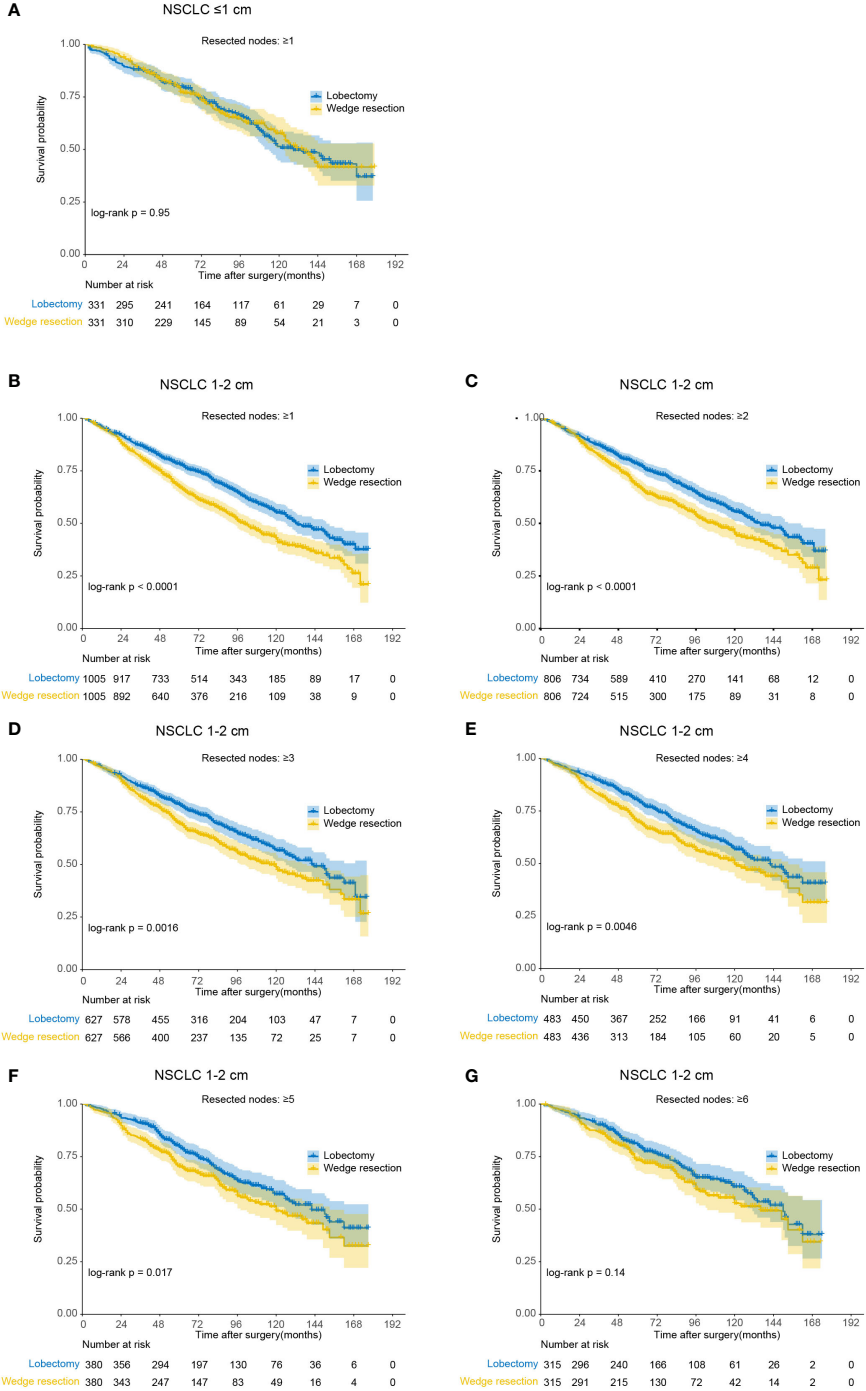
Kaplan-Meier analysis of overall survival between wedge resection and lobectomy for non-small cell lung cancer (NSCLC) with different number of nodes resected (after matching). **(A)** NSCLC ≤1 cm with ≥1 nodes resected; **(B)** NSCLC 1-2 cm with ≥1 nodes resected; **(C)** NSCLC 1-2 cm with ≥2 nodes resected; **(D)** NSCLC 1-2 cm with ≥3 nodes resected; **(E)** NSCLC 1-2 cm with ≥4 nodes resected; **(F)** NSCLC 1-2 cm with ≥5 nodes resected; **(G)** NSCLC 1-2 cm with ≥6 nodes resected.

For NSCLC ≤1cm, patients derived similar CSS and OS under wedge resection or lobectomy with no less than 1 nodes resected (**Figure 4** and **Table 3**). Notably, the lobectomy group have significantly more nodes resected over wedge resection group in the balanced population (median number, 7 versus 3, *p*<0.001). For NSCLC 1-2cm, wedge resection generated inferior CSS and OS versus lobectomy when less than 6 nodes were examined. In the subset including NSCLC with 6 or more nodes examined, the two procedures showed similar long-term outcomes (*log-rank p*=0.14). In the multivariable analysis, the increased risk of deaths in patients undergoing wedge resection descended gradually along with the increase of the number of resected lymph nodes (**Table 3**). Beyond 6 nodes, the difference on OS did not reach statistically significance (HR, 1.21, 95% CI: 0.93-1.59). The median number of resected lymph nodes between the two groups was similar as well (10 versus 9, *p*=0.544). Analysis on CSS draw similar conclusions as that on OS.

**Table 3:** Univariable and multivariable analysis of cause-specific survival and overall survival among patients undergoing wedge resection or lobectomy with different number of lymph nodes resected (after matching).

	Cause-Specific Survival				Overall Survival				Number of lymph nodes examined, mean (median)		
	Univariable analysis		Multivariable analysis		Univariable analysis		Multivariable analysis				
Size	HR (95% CI)	*p* value	HR (95% CI)	*p* value	HR (95% CI)	*p* value	HR (95% CI)	*p* value	Lobectomy	Wedge resection	*p* value
0-1 cm											
All	1.34 (1.03-1.74)	0.030	1.36 (1.04-1.77)	0.023	1.36 (1.14-1.63)	0.001	1.41 (1.18-1.68)	<0.001	8.9 (7)	2.4 (1)	<0.001
≥1 nodes	1.19 (0.82-1.74)	0.362	1.22 (0.84-1.78)	0.301	0.99 (0.77-1.28)	0.947	1.03 (0.80-1.34)	0.815	8.9 (7)	4.7 (3)	<0.001
1-2 cm											
All	1.88 (1.63-2.17)	<0.001	1.91 (1.66-2.21)	<0.001	1.69 (1.54-1.86)	<0.001	1.72 (1.56-1.89)	<0.001	8.7 (7)	2.8 (1)	<0.001
≥1 nodes	1.68 (1.36-2.07)	<0.001	1.67 (1.35-2.06)	<0.001	1.47 (1.28-1.69)	<0.001	1.45 (1.26-1.66)	<0.001	8.8 (7)	5.2 (3)	<0.001
≥2 nodes	1.50 (1.19-1.90)	0.001	1.51 (1.19-1.91)	<0.001	1.39 (1.19-1.63)	<0.001	1.37 (1.17-1.61)	<0.001	9.3 (8)	6.3 (4)	<0.001
≥3 nodes	1.40 (1.07-1.83)	0.014	1.41 (1.08-1.85)	0.012	1.34 (1.12-1.61)	0.002	1.34 (1.11-1.61)	0.002	9.8 (8)	7.5 (6)	<0.001
≥4 nodes	1.41 (1.04-1.93)	0.028	1.43 (1.05-1.95)	0.023	1.35 (1.10-1.67)	0.005	1.35 (1.10-1.67)	0.005	10.3 (9)	8.8 (7)	<0.001
≥5 nodes	1.45 (1.03-2.04)	0.035	1.45 (1.03-2.05)	0.036	1.33 (1.05-1.69)	0.017	1.32 (1.05-1.68)	0.020	10.9(9)	10.1 (8)	0.090
≥6 nodes	1.25 (0.85-1.84)	0.267	1.24 (0.84-1.82)	0.285	1.23 (0.94-1.61)	0.137	1.21 (0.93-1.59)	0.159	11.5 (10)	11.2 (9)	0.544

CI, confidence interval; HR, hazard ratio.

## Discussion

Our study reveals that wedge resection led to worse OS than lobectomy for node-negative NSCLC ≤2 cm in the population. These two procedures were comparable when one or more nodes for lesions ≤1 cm and six or more nodes for lesions 1-2 cm were resected. For lesions 1-2 cm and receiving lobectomy, more nodes resected conferred statistically significant increase on OS, while for lesions ≤1 cm not.

The choice of surgical procedures for NSCLC ≤2 cm have gained remarkable attentions recently. The usage of high-solution CT scanning and the spread of lung cancer screening led to the high detection of those small pulmonary nodules. Suitable surgical treatment can effectively prevent the recurrence and achieve the goal of cure. Recently published results from JCOG0804 study suggested that the 5-year relapse-free survival of sublobar resection was 99.7% for ground-glass opacity (GGO) dominant peripheral lung cancer with maximum diameter less than 2 cm and consolidation/tumor ratio (CTR) ≤0.25 on the preoperative thin-section CT (9). For tumors ≤2 cm and CTR >0.5, JCOG0802 study showed that segmentectomy had improved OS over lobectomy, although segmentectomy had similar relapse-free survival and higher local relapse rate (10). The ongoing JCOG1211 study would explore the efficacy of segmentectomy for clinical lung cancer ≤2 cm with a CTR from 0.25 to 0.5 (11). Besides, National Comprehensive Cancer Network guidelines recommended sublobar resection for peripheral nodules ≤2 cm with adenocarcinoma *in situ* histology, or ≥50% ground-glass appearance on CT or a doubling time of ≥400 days (12). However, the JCOG trials had its limitations in clinical decision-making. Recommendations based on the combination of whole tumor size and CTR may be conflicting when considering the solid size (13).

Wedge resection was an essential choice of procedures when treating small-sized NSCLC. We found that wedge resection was performed for 22.9% patients with NSCLC less than 2 cm diagnosed between 2004 and 2015 in SEER database (lobectomy 71.4%, segmentectomy 5.7%). However, our study revealed that after propensity score matching, wedge resection resulted in 41% and 72% increase of death risk over lobectomy for lesions less than 1 cm and 1-2 cm, respectively. Therefore, it has clinical significance for wedge resection to achieve similar long-term outcomes compared with lobectomy. Considering the higher nonexamination rate of lymph node for wedge resection (47.3%) over lobectomy (3.5%), adequate lymph node resection can narrow the survival gap between those two procedures. As our prior study demonstrated, lymph node resection can improve long-term survival of wedge resection irrespective of the tumor size ≤1 cm or 1-2 cm (5). Increased number of lymph nodes resected were associated with more accurate pathological staging and better local control, which can guide the administration of adjuvant therapy and improve long-term survival after the curative intent operation (14). Wolf’s study showed that sublobar resection (wedge resection, 130/154) with lymph nodes sampling had an similar OS and recurrence-free survival to that of lobectomy for NSCLC ≤2 cm (15). Ajmani’s study showed that for cT1-2N0M0 NSCLC undergoing wedge resection, the rate of nodal upstaging rate increased from 4.4% for patients with 1-5 nodes harvested to 8.1% for patients with ≥10 nodes harvested (16). Unlike the results of our analysis, Stiles’s study showed that the inferiority of sublobar resection over lobectomy disappeared when nine or more nodes resected (8). The study enrolled patients aged over 66 years and received wedge resection or segmentectomy between 2007 and 2012 from SEER-Medicare database. That may be the reason leading to the difference.

The study has several limitations. First, selecting bias existed due to the retrospective nature of the present study, although propensity score matching was performed to minimize that limitation. Second, several clinical factors such as pulmonary function, smoking history and main comorbidities can impact the choice of procedures and OS. However, those factors were not available in the SEER database. Third, besides the number of lymph nodes, the number of stations examined had prognostic effect during wedge resection. The prior study showed that compared to patients with only mediastinal lymph nodes (N2) or only one station of regional lymph nodes (N1) evaluated, those who had N1 stations or more than one N1 stations harvested achieved better OS and recurrence-free survival (17). Regrettably, SEER database does not record respective number of resected N1 or N2 nodes.

In conclusion, our study revealed the inferiority of wedge resection versus lobectomy for NSCLC ≤1 cm or 2 cm in the population. However, wedge resection plus adequate lymph node resection can generate equivalent clinical outcomes to lobectomy.

## Data availability statement

Publicly available datasets were analyzed in this study. This data can be found here: https://seer.cancer.gov/.

## Author contributions

Study concepts: HD, GJ, HW. Study design: HD, NS, HW. Data acquisition: HD, NS, PZ. Quality control of data and algorithms: NS, GJ, HW. Data analysis and interpretation: HD, NS. Statistical analysis: HD, NS. Manuscript preparation: HD. Manuscript editing: HD. Manuscript review: GJ, HW. All authors contributed to the article and approved the submitted version.

## Funding

This work was supported by Shanghai Sailing Program of Shanghai Science and Technology Committee (grant number: 22YF1437300) and Young Eagle Plan of Shanghai Pulmonary Hospital (grant number: fkcy1905).

## Conflict of interest

The authors declare that the research was conducted in the absence of any commercial or financial relationships that could be construed as a potential conflict of interest.

## Publisher’s note

All claims expressed in this article are solely those of the authors and do not necessarily represent those of their affiliated organizations, or those of the publisher, the editors and the reviewers. Any product that may be evaluated in this article, or claim that may be made by its manufacturer, is not guaranteed or endorsed by the publisher.

## Supplementary material

Supplementary Figure 1 Kaplan-Meier analysis of survival between wedge resection and lobectomy before matching. **(A)** Cause-specific survival for non-small cell lung cancer (NSCLC) ≤1 cm; **(B)** overall survival for NSCLC ≤1 cm; **(C)** cause-specific survival for NSCLC 1-2 cm; **(D)** overall survival for NSCLC 1-2 cm.

## Abbreviations

CT, computed tomography; HR, hazard ratio; LOESS, locally estimated scatterplot smoothing; NSCLC, non-small cell lung cancer; OS, overall survival; SEER, Surveillance, Epidemiology, and End Results.
