# Supplementary material for: Wedge resection plus adequate lymph nodes resection is comparable to lobectomy for small-sized non-small cell lung cancer
Source: Front Oncol. 2022 Nov 11;12:1022904. doi: 10.3389/fonc.2022.1022904 (PMC9691685; doi:10.3389/fonc.2022.1022904)
Supplement: Supplementary file 1 [file DataSheet_1.docx]

| **Supplemental Table1**. Baseline Characteristics of Patients With NSCLC 1 cm or Less or 1-2 cm Undergoing Wedge Resection or Lobectomy (n=5072, after matching) | | | | | | |
| --- | --- | --- | --- | --- | --- | --- |
| Characteristic | NSCLC ≤1 cm (n=1308) | | *p* value | NSCLC 1-2 cm (n=3764) | | *p* value |
|  | Lobectomy (n=654) | Wedge resection (n=654) |  | Lobectomy (n=1882) | Wedge resection (n=1882) |  |
| Sex, female | 392 (59.9) | 411 (62.8) | 0.307 | 1080 (57.4) | 1079 (57.3) | 1.000 |
| Age, >65 years | 436 (66.7) | 430 (65.7) | 0.77 | 1344 (71.4) | 1337 (71.0) | 0.829 |
| Race, nonwhite | 85 (13.0) | 94 (14.4) | 0.52 | 234 (12.4) | 239 (12.7) | 0.844 |
| Marriage |  |  | 0.504 |  |  | 0.996 |
| Married | 379 (58.0) | 369 (56.4) |  | 987 (52.4) | 987 (52.4) |  |
| Unmarried | 253 (38.7) | 255 (39.0) |  | 819 (43.5) | 818 (43.5) |  |
| Unknown | 22 ( 3.4) | 30 ( 4.6) |  | 76 ( 4.0) | 77 ( 4.1) |  |
| Histologic type |  |  | 0.987 |  |  | 0.889 |
| Adenocarcinoma | 472 (72.2) | 473 (72.3) |  | 1241 (65.9) | 1229 (65.3) |  |
| Squamous carcinoma | 137 (20.9) | 135 (20.6) |  | 482 (25.6) | 495 (26.3) |  |
| Others | 45 ( 6.9) | 46 ( 7.0) |  | 159 ( 8.4) | 158 ( 8.4) |  |
| Grade |  |  | 0.579 |  |  | 0.982 |
| Well differentiated | 229 (35.0) | 213 (32.6) |  | 412 (21.9) | 417 (22.2) |  |
| Moderately differentiated | 213 (32.6) | 220 (33.6) |  | 814 (43.3) | 804 (42.7) |  |
| Poorly differentiated | 147 (22.5) | 141 (21.6) |  | 497 (26.4) | 499 (26.5) |  |
| Undifferentiated | 3 ( 0.5) | 6 ( 0.9) |  | 24 ( 1.3) | 28 ( 1.5) |  |
| Unknown | 62 ( 9.5) | 74 (11.3) |  | 135 ( 7.2) | 134 ( 7.1) |  |
| Site |  |  | 0.958 |  |  | 0.829 |
| Upper lobe | 439 (67.1) | 434 (66.4) |  | 1216 (64.6) | 1204 (64.0) |  |
| Middle lobe | 26 ( 4.0) | 27 ( 4.1) |  | 74 ( 3.9) | 78 ( 4.1) |  |
| Lower lobe | 182 (27.8) | 184 (28.1) |  | 577 (30.7) | 580 (30.8) |  |
| Others | 7 ( 1.1) | 9 ( 1.4) |  | 15 ( 0.8) | 20 ( 1.1) |  |
| Tumor size, mean±SD, cm | 0.86±0.20) | 0.83±0.21 | 0.024 | 1.62±0.29) | 1.57±0.29) | <0.001 |
| Lymph nodes examined, median (IQR) | 7 (4-12) | 1 (0-3) | <0.001 | 7 (4-11) | 1 (0-4) | <0.001 |
| Follow-up time, median (range), months | 68 （2-178） | 60 （2-179） | 0.001 | 69 (2-178) | 55 (2-179) | <0.001 |
| IQR, interquartile range; NSCLC, non-small cell lung cancer. | |  |  |  |  |  |

**Supplemental Table2**. Univariable and Multivariable Analysis of Cause-Specific Survival Among Patients Undergoing Lobectomy With Different Number of Lymph Nodes Resected (0 as reference)

| Size | | Univariable analysis | |  | Multivariable analysis | |
| --- | --- | --- | --- | --- | --- | --- |
|  |  | HR (95% CI) | *p* value |  | HR (95% CI) | *p* value |
| 0-1 cm |  |  |  |  |  |  |
|  | ≥1 nodes | 0.54 (0.27-1.05) | 0.070 |  | 0.52 (0.26-1.03) | 0.059 |
|  | 1-3 nodes | 0.58 (0.28-1.21) | 0.146 |  | 0.55 (0.26-1.16) | 0.114 |
|  | 4-9 nodes | 0.52 (0.26-1.05) | 0.067 |  | 0.51 (0.25-1.02) | 0.058 |
|  | ≥10 nodes | 0.53 (0.26-1.08) | 0.080 |  | 0.52 (0.25-1.06) | 0.073 |
| 1-2 cm |  |  |  |  |  |  |
|  | ≥1 nodes | 0.47 (0.37-0.60) | <0.001 |  | 0.51 (0.40-0.64) | <0.001 |
|  | 1-3 nodes | 0.64 (0.49-0.82) | 0.001 |  | 0.68 (0.53-0.88) | 0.004 |
|  | 4-9 nodes | 0.46 (0.36-0.59) | <0.001 |  | 0.49 (0.38-0.63) | <0.001 |
|  | ≥10 nodes | 0.41 (0.31-0.52) | <0.001 |  | 0.43 (0.33-0.55) | <0.001 |
| CI, confidence interval; HR, hazard ratio. | | | |  |  |  |

**Supplemental Table3**. Multivariable Analysis of Overall Survival among 1-2 cm NSCLC Undergoing Lobectomy (0 as reference)

| Number of lymph nodes resected | HR (95% CI) | *p* value |  |
| --- | --- | --- | --- |
| 1 | 0.781 (0.593-1.03) | 0.078 |  |
| 2 | 0.551 (0.401-0.758) | <0.001 |  |
| 3 | 0.493 (0.347-0.7) | <0.001 |  |
| 4 | 0.401 (0.279-0.577) | <0.001 |  |
| 5 | 0.44 (0.303-0.639) | <0.001 |  |
| 6 | 0.388 (0.266-0.567) | <0.001 |  |
| 7 | 0.444 (0.309-0.637) | <0.001 |  |
| 8 | 0.391 (0.273-0.559) | <0.001 |  |
| 9 | 0.441 (0.315-0.617) | <0.001 |  |
| 10 | 0.451 (0.325-0.626) | <0.001 |  |
| 11 | 0.55 (0.408-0.741) | <0.001 |  |
| 12 | 0.346 (0.249-0.482) | <0.001 |  |
| 13 | 0.529 (0.388-0.721) | <0.001 |  |
| 14 | 0.378 (0.271-0.528) | <0.001 |  |
| 15 | 0.427 (0.302-0.605) | <0.001 |  |
| CI, confidence interval; HR, hazard ratio; NSCLC, non-small cell lung cancer. | | | |
