# Supplementary figures and images for: Wedge resection plus adequate lymph nodes resection is comparable to lobectomy for small-sized non-small cell lung cancer
Source: Front Oncol. 2022 Nov 11;12:1022904. doi: 10.3389/fonc.2022.1022904 (PMC9691685; doi:10.3389/fonc.2022.1022904)

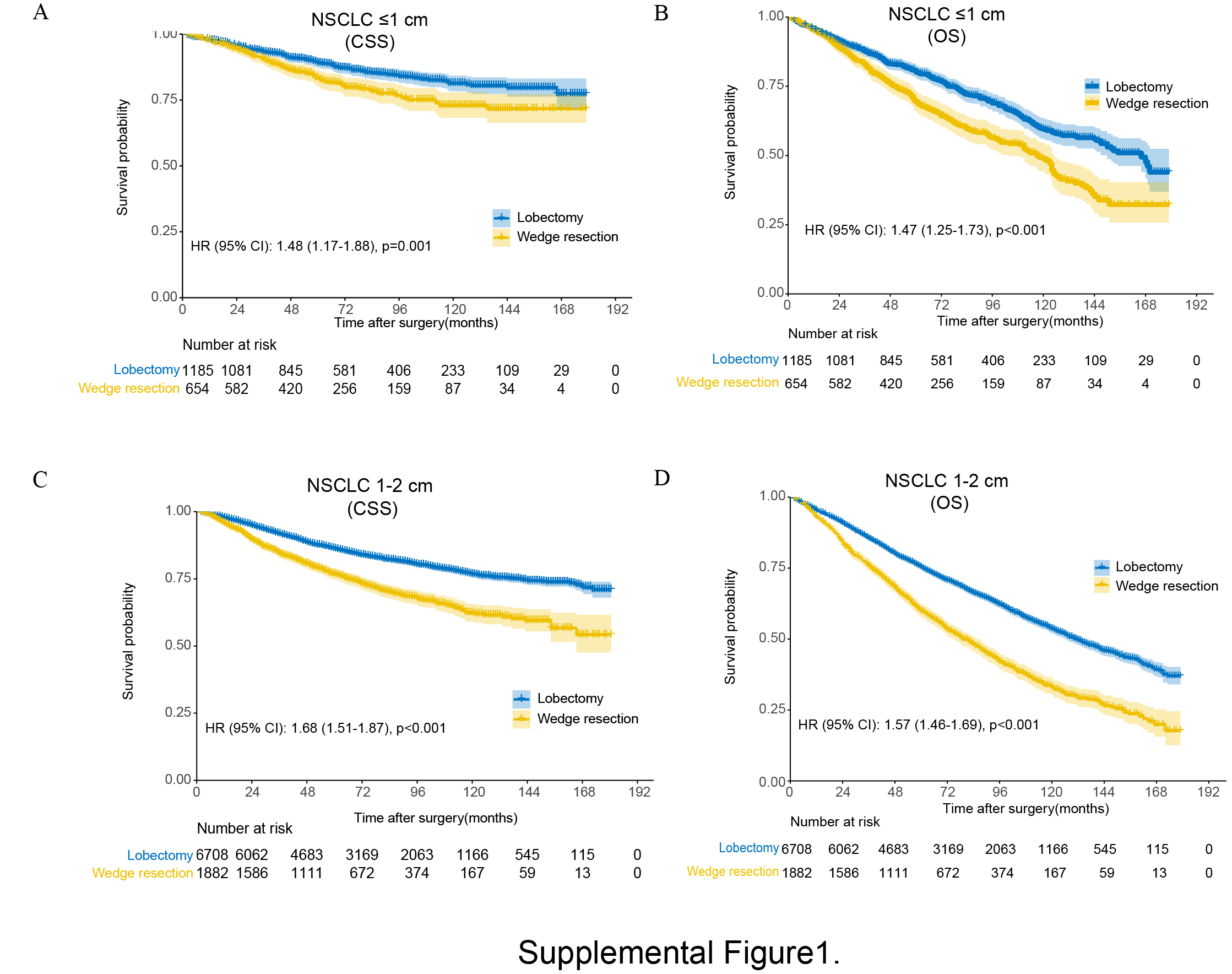

Supplement: Supplementary Figure 1 — Kaplan-Meier analysis of survival between wedge resection and lobectomy before matching. (A) Cause-specific survival for non-small cell lung cancer (NSCLC) ≤1 cm; (B) overall survival for NSCLC ≤1 cm; (C) cause-specific survival for NSCLC 1-2 cm; (D) overall survival for NSCLC 1-2 cm. [file Image_1.jpeg]
